# Regenerative Therapies in Dry Eye Disease: From Growth Factors to Cell Therapy

**DOI:** 10.3390/ijms18112264

**Published:** 2017-10-28

**Authors:** Antonio J. Villatoro, Viviana Fernández, Silvia Claros, Cristina Alcoholado, Manuel Cifuentes, Jesús Merayo-Lloves, José A. Andrades, José Becerra

**Affiliations:** 1 Department of Cell Biology, Genetics and Physiology, University of Málaga, IBIMA, 29071 Málaga, Spain; antoniojvillatoro@gmail.com (A.J.V.); vivianafdezgen@yahoo.com (V.F.); silviacg@uma.es (S.C.); alcoholado.c@gmail.com (C.A.); mcifuentes@uma.es (M.C.); becerra@uma.es (J.B.); 2 Networking Research Center on Bioengineering, Biomaterials and Nanomedicine, (CIBER-BBN), 29071 Málaga, Spain; 3 Fundación de Investigación Oftalmológica, 33012 Oviedo, Spain; merayo@fio.as; 4 Laboratory of Bioingeneering and Tissue Regeneration, Andalusian Center for Nanomedicine and Biotechnology-BIONAND, 29590 Málaga, Spain

**Keywords:** mesenchymal stem cell, allogenic cell therapy, growth factor, lacrimal gland, dry eye, keratoconjunctivitis sicca, regenerative medicine

## Abstract

Dry eye syndrome is a complex and insidious pathology with a high level of prevalence among the human population and with a consequently high impact on quality of life and economic cost. Currently, its treatment is symptomatic, mainly based on the control of lubrication and inflammation, with significant limitations. Therefore, the latest research is focused on the development of new biological strategies, with the aim of regenerating affected tissues, or at least restricting the progression of the disease, reducing scar tissue, and maintaining corneal transparency. Therapies range from growth factors and cytokines to the use of different cell sources, in particular mesenchymal stem cells, due to their multipotentiality, trophic, and immunomodulatory properties. We will review the state of the art and the latest advances and results of these promising treatments in this pathology.

## 1. Introduction

Dry eye disease (DED) has been defined as a multifactorial disease of the ocular surface characterized by a loss of homeostasis of the tear film, and is accompanied by ocular symptoms in which tear film instability and hyperosmolarity, ocular surface inflammation and damage, and neurosensory abnormalities play etiological roles [1]. This pathology is also often secondary to a multisystem autoimmune disease such as Sjögren’s Syndrome, rheumatoid arthritis, systemic lupus erythematosus, etc., and is a source of frustration for professionals and patients [2,3,4,5,6,7].

One study found that the prevalence of DED ranged from approximately 5% to 50% [8], with higher rates among women and the elderly [4,6,9]. Consequently, DED is an important public health problem that leads to a sanitary high cost, hinders the performance of the activities of daily living, and therefore decreases quality of life [2,3,4,5,6,7].

Recently, it was agreed by the Tear Film and Ocular Surface Society (TFOS) International Dry Eye Workshop (DEWS) that tear hyperosmolarity and tear instability are the core drivers of DED. This allowed two major subtypes to be defined: evaporative dry eye (EDE), where tear hyperosmolarity is the result of an excessive evaporation of the tear film in the presence of normal lacrimal function; and aqueous-deficient dry eye (ADDE), where hyperosmolarity results from a reduced lacrimal secretion in the presence of a normal rate of tear evaporation [10].

Although its pathophysiology is still unclear, the Committee for the International Dry Eye Workshop highlighted the crucial roles of hyperosmolarity and inflammation in DED [1]. In certain conditions, there is an increase in the osmolarity of the tear film, either due to poor tear function or to excessive evaporation of the aqueous tear component, with normal lacrimal secretory function [1,2,5]. This triggers a hyperosmotic state of the ocular surface, which initiates an inflammatory response involving both innate and adaptive immune systems [1,4,5,8].

Despite the multifactorial nature of DED, this disease can be chronically self-maintained through a vicious cycle [10], where the epithelial damage secondary to the hyperosmolar state causes exposure and chronic stimulation of corneal nerve endings. Reduction in corneal sensitivity promotes neurogenic stress, contributing to the impairment of ocular surface homoeostasis and the release of proinflammatory factors responsible for greater damage to the ocular surface and to the gland itself [4,8,10].

An inflamed lacrimal gland may produce abnormal tears containing proinflammatory cytokines, disrupting the ocular surface, activating angiogenesis and lymphangiogenesis, and exacerbating the inflammatory response. This perpetuates a chronic inflammatory process responsible for the ocular surface damage, visual impairment, and other associated symptoms [1,4,10].

Squamous metaplasia of the epithelial cells on the ocular surface occurs, with a gradual loss of conjunctival goblet cells and an increase in inflammatory cells as well as an increase in the number of apoptotic epithelial cells [4,8,10].

Currently, there is no cure for dry eye, and the treatments are directed towards improving the symptoms in order to break the vicious circle of DED and to prevent chronicity and progression of the disease [1,2,10].

The mainstay of conventional therapy is the application of artificial tears that increase moisture on the ocular surface and provide additional lubrication [11]. Other pharmacological approaches—anti-inflammatory and topical immunosuppressory—are used to improve the symptoms of chronic inflammation [10]. Steroids are the most commonly prescribed short-term treatment for managing DED-associated inflammation, but their long-term use is not recommended [1,10]. Cyclosporine A is an immunosuppressive peptide derived from fungal origin, and is used as an anti-inflammatory topical drop for DED treatment. However, adverse ocular events have been reported [2,10,12]. In recent years, new regenerative strategies have emerged that have made possible a qualitative advance in the management of this pathology.

## 2. Hemoderivatives

The use of drops of different blood products in the DED treatment and other pathologies of the ocular surface has resulted in a remarkable advance in the management of severe cases refractory to conventional therapy [13,14,15]. Currently, the most common preparations are the use of autologous or allogeneic serum drops, platelet-derived plasma products, and umbilical cord blood serum [16,17].

### 2.1. Autologous Serum (AS)

Serum is the liquid fraction of whole blood that is collected after the blood is allowed to clot. The clot can be removed by centrifugation and the resulting supernatant—the serum—is prepared for use as drops [15]. Autologous serum (AS) application was employed in 1975 to treat ocular alkali injuries [18]. In 1984, its first successful use in patients with dry eye was described [19]. However, from the works of Tsubota et al. [20,21] in 1999, AS application gained widespread acceptance as an adjuvant therapy in different ocular surface disorders [3,15,22,23].

The therapeutic advantages of AS use as a substitute for tears are given by their similarity in certain characteristics, such as pH, osmolality, and biomechanical characteristics. AS fulfills a lubricating function and performs anti-inflammatory, antimicrobial, and epitheliotrophic functions through certain biomolecules of its composition similar to natural tears [13,23] (Table 1).

In AS, composition is emphasized; various substances are present which also exist in normal tears, including growth factors such as transforming growth factor (TGF-β), platelet-derived growth factors (PDGF), epidermal growth factor (EGF), nerve growth factor, and insulin-like growth factor 1; neurotrophic factors (substance P); cytokines; bacteriostatic factors (lactoferrin, lysozyme, immunoglobulins); fibronectin and vitamin A and E [13,23,24] (Table 1). Some of them have an increased concentration with respect to natural tears, such as vitamin A, lysozyme, TGF-β, and fibronectin, and some components are present in lesser concentrations, such as immunoglobulin A, epithelial EGF, and vitamin C [15,23,24].

TGF-β is known to have dose-dependent antiproliferative properties, and its average levels are usually five times higher in AS than in natural tears, so it is usually used at 20% dilution to prevent the potentially harmful effect of avoiding possible retardation of epithelial wound healing. Nonetheless, dilution may reduce the concentration of other beneficial factors—particularly EGF and fibronectin, which are proven to support the proliferation and migration of corneal epithelial cells [3,15,23,24,25].

Despite many clinical studies demonstrating their efficacy, there is no standard protocol for obtaining AS [16,17]. Individual particularities, patient health status, collection of whole blood, production protocol, dilution, storage, and treatment regimens, have been described as factors that affect the composition and efficacy of the product, leading to variability in the results [7,15,26,27,28].

However, the presence of leucocytes during the AS preparation procedure increases the level of pro-inflammatory cytokines (interleukin 6 (IL-6), IL-1β, and tumor necrosis factor-α (TNF-α), etc.), added to the presence of immunoglobulins and complement, may be deleterious for many patients suffering from immunological alterations [29].

Although some growth factors (e.g., EGF, TGF-β, and insulin-like growth factor 1 (IGF-1)) are relatively stable, neurotrophic factors as substance P (SP) and calcitonin gene-related peptide (CGRP) significantly degraded at −15 °C in 6 weeks and at +4 °C in 24 h [15]. Thus, AS storage must be carried out in freezer at −20 °C, and it must be thawed before use and kept in in the refrigerator at +4 °C if it is to be stored for 24 h to a week. The serum eye drops must be used within three months of the date of production [4,23,27]. It is important that vials containing AS be kept away from light to avoid the degradation of vitamin A [15].

The AS tears regimen of daily application goes from hourly up to three times a day [13,15,26]. Preservatives are usually not added to AS, thus reducing the risk of preservative-induced toxicity. However, a lack of preservatives theoretically increases the risk of ocular infections [14,23,26].

Few studies have directly compared clinical outcomes of different concentrations of AS [13,15,30]. Most published studies have reported the use of 20% AS eye drops for treating a number of ocular surface conditions, mainly in dry eye, where AS suppresses apoptosis in the ocular surface epithelium and increases goblet cell density in dry eye [23,30]. Nevertheless, its potential benefits have been questioned by a recent meta-analysis [15].

Undiluted serum was more effective in epithelial cell migration and epithelial healing in postoperative corneal epithelial defect following various ocular surgeries, probably because of the higher concentration of fibronectin, shortening the healing time and decreasing the risk of chronic epithelial defect and complications [14,28].

The TFOS DEWS II Management and Therapy Report shows a table with results from 14 clinical studies on the efficacy of autologous serum in DED. Among these studies there is substantial variation for production parameters, endpoints, dose frequency, and treatment duration. Sixty to eighty percent of patients showed positive responses [31].

### 2.2. Allogeneic Serum (ALS)

ALS from healthy blood donors was used when a patient’s own serum was unsuitable, unavailable, or where repeated blood sampling was not possible, including patients with viral infection, septicemia, severe anemia, and elderly patients with multiple systemic diseases [7,17,26].

ALS use offers an additional advantage in some immune-mediated pathologies with large inflammatory and systemic component, where the direct transfer of AS containing elevated levels of pro-inflammatory cytokines to the eye should be avoided [7,32].

On the other hand, ALS would allow the production of large quantities of tears, greatly improving the logistics of the treatment and making it possible to standardize and screen the composition of cytokines, anti-inflammatory, and epitheliotrophic components to improve their efficacy [7].

### 2.3. Platelet-Rich Plasma (PRP)

Under the generic term PRP is included a variety of products and denominations derived from the patient’s own blood, which can be obtained by centrifugation to obtain a plasma fraction with a platelet concentration higher than that in the circulating blood [33]. Platelets can be artificially activated and release their contents housed in alpha granules, rich in a large pool of proteins and factors including EGF, PDGF, TGF-β, secrete vascular endothelial growth factor (VEGF), IGF-1, hepatic growth factor (HGF), nerve growth factor (NGF), and platelet factor 4 (PF-4) involved in the wound healing process of the cornea and conjunctival surface [34,35] (Table 1).

Depending on their preparation, composition, and especially the concentration of platelets and the presence or absence of leucocytes, different products are obtained: PDGF, plasma rich in growth factors (PRGF), plasma rich in platelet and growth factors (PRPGF), platelet concentrate (PC), leukocyte-rich platelet-rich plasma (LR-PRP), leukocyte-poor platelet-rich plasma (LP-PRP), among others [13,36,37].

Currently, there are more than 40 different preparation methods, which makes it difficult to compare different scientific results in terms of both efficacy and safety [13,35,38]. Regarding the industrialization of PRGF eye drops, there are studies of preservation and biological activity for 3 months in human use, with good outcomes in the care for patients with severe dry eye that do not respond to conventional therapy [39], with special success in neutrophic cases [40]. Finally, PRGF could be used to support the growth of limbal stem cells [41,42].

PRP cellular composition defines the concentrations of growth factor and catabolic cytokine. The platelet concentration is positively correlated with all growth factors, increasing anabolic signaling [36,37].

Leukocytes strongly influence the quality of PRPs. Leukocytes increased catabolic signaling molecules like matrix metallopeptidase 9 (MMP-9) that are strongly correlated with the leukocyte concentration. There is a direct correlation between leucocytes with PDGF and the VEGF concentration, while it is negatively correlated with fibroblast growth factor (FGF) [37].

The role of non-platelet components of whole blood contributes to their biological activity—in particular, red and white blood cells may be detrimental by participating in unwanted inflammatory reactions. In spite of the possible negative pro-inflammatory effect caused by the presence of leukocyte [29,38], other studies suggest that the non-platelet cellular components are important for optimal platelet function, including thrombin generation leading to robust coagulation, growth factor release, and the resulting capacity of the serum to stimulate cell proliferation [36].

Different investigations have evaluated the safety and efficacy of the use of different PRP preparations in the treatment of dry eye, demonstrating an improvement in tear film quality and the severity of symptoms, even in patients previously treated with AS [38,39]. PRP has certain contraindications to its use, such as serious cardiac disease in the extraction phase, active bacterial infections, and a history of certain viral infectious diseases (B hepatitis, HIV, etc.) [35].

Therefore, depending on the clinical application, modifying the PRP preparation method should be considered based on their ability to concentrate platelets and leukocytes with sensitivity to pathologic conditions essential to achieve better clinical results.

### 2.4. Umbilical Cord Blood Serum (UCS)

Like peripheral blood serum, UCS contains a high concentration of tear components. Compared to blood serum, concentrations of EGF and TGF-β are three and two times higher, respectively. UCS has higher NGF and SP and lower IGF-1 and vitamin A, but it is higher than the concentration in normal tears [29,43]. UCS shows a bacteriostatic effect because it contains antibacterial agents such as immunoglobulin G, lysozyme, and complement [39] (Table 1).

UCS eye drops are recommended to use at 20% concentration, and are usually instilled four to six times per day. They must be stored at −20 °C for 3 to 6 months [29,43]. They have been used to treat various ocular surface diseases, including severe dry eye with or without Sjögren’s syndrome, ocular complications in graft-versus-host disease, persistent epithelial defects, neurotrophic keratopathy, recurrent corneal erosions, ocular chemical burn, and surface problems after corneal refractive surgery [13,43,44].

Compared with AS eye drops, UCS eye drops have been more effective in decreasing symptoms and have epitheliotrophic effects, increasing goblet cell density in severe dry eye syndrome [29].

A large amount of sample can be drawn from the umbilical vein at the time of delivery, so that the requirement for several patients can be met at the same time. The risk of allergies and the possibility of transmitting parenteral diseases must be evaluated [44].

## 3. Stem Cell Therapy

In the last decade, there has been an emerging interest in stem cell therapy for different pathologies, including ocular diseases [45,46,47,48]. Among all cell candidates to be employed, mesenchymal stem cells or multipotent stromal cells (MSCs) have been the most interesting for researchers [46,49].

### 3.1. MSCs (Auto/Allogeneic)

MSCs are currently proposed as cell therapy for many diseases, particularly those with an inflammatory and immunomediated component. There are over 740 clinical trials now listed at www.clinicaltrials.gov using MSCs. Autologous and allogenic cell therapies are now ongoing.

MSCs are a group of fibroblast-like self-renewing, non-hematopoietic, multipotent progenitor cells, and are ontogenically derived from the embryonic layer of the mesoderm. The International Society for Cellular Therapy has suggested minimal criteria to define the MSC: they are plastic-adherent, must present a certain surface molecule profile and be able to differentiate to at least three mesenchymal lineages (osteogenesis, adipogenesis, and chondrogenesis) [50]; in addition, they must maintain their immunomodulatory potential [51]. Due to a lack of major histocompatibility complex II (MHC-II) expression and co-stimulatory molecules (such as CD40, CD80, and CD86), they can be used allogenetically since they escape the recognition and action of T cells and natural killer (NK) cells [52].

They fulfil a function as a reservoir of undifferentiated cells for the regeneration of the tissues where they are located, having been isolated in diverse adult or extraembryonic tissues [53,54]. They present the migration and homing capacity to the site of the lesion in response to the cytokines, chemokines, and growth factors released [55,56]. In addition, they do not raise ethical issues and have very low tumorigenesis potential [57,58,59,60].

Different mesenchymal stem-like cell populations have been identified in the eye: basal limbus, corneal stroma, trabecular meshwork, choroids, and periorbital fat [47,61,62]. MSCs can differentiate both towards mesenchymal lineages and other germ lines [58,59], among them different cell types present in the corneal surface such as epithelial, stromal, and endothelial [55,60,63].

Actually, it is well established that the ability to modulate the immune system plays a fundamental role in almost all the therapeutic effects attributed to these cells, rather than their capacity of differentiation in different cell lineages [54,60,64,65]. This property is carried out through the release of a large variety of bioactive substances with autocrine and paracrine effects, encompassed under the concept of the secretome [56,66]. There are included a huge variety of molecules, including proteins, growth factors, antioxidants, proteasomes, microvesicles, and exosomes, which target a multitude of biological targets (pleiotropic effect) [67,68], and are responsible for different effects: production of extracellular matrix, antiapoptotic, antifibrotic, chemoattractive, neuroprotective, morphogenic, angiogenic, antimicrobial, immunomodulatory, etc. [56,69,70]. The composition of the secretome and its immunomodulatory capacity varies with the species, source method of manufacture, medication, and microenvironment where the MSCs are homeing [49,54,71].

The immunomodulatory effect of MSCs is exerted on both the innate and adaptive immune response, through different mechanisms such as direct cell-to-cell contact and the secretion of different soluble substances in their secretome [65]. In particular, the indolamine 2,3-dioxygenase (IDO), prostaglandin E2 (PGE2), TGF-β, HGF, nitric oxide (NO), IL-1, IL-6, and interleukin 1 receptor antagonist, among others [64,65,72]. However, their immunomodulatory mechanism is not restricted to soluble factors. Recently, it has been shown that the exosomes excreted by MSCs modulate the inflammatory response, in addition to other functions via direct action on resident cell targets [67,72,73].

Regarding innate immunity, MSCs have demonstrated the ability to modulate different types of cells that constitute this first line of defense, such as macrophages, neutrophils, dendritic cells, and NK [54,65,72,74]. Regarding adaptive immunity, they demonstrate a very interesting aspect of immunomodulation, due to their broad action capacity on Th1, Th2, and Th17 responses [54,65,75,76].

It is considered that one of the major MSCs’ immunomodulation mechanisms is the regulation of T cells—both CD4+ and CD8+—by cell-to-cell contact and inhibitory molecules of their secretome [54,67,76]. They are also able to act on B cells by modifying their activation, proliferation, chemotactic response, and differentiation to becoming antibody-secreting plasma cells [54,65,75,76].

The immunomodulatory capacity of MSCs is also complemented by their important potential to promote the generation and maintenance of the activity of different types of regulatory T cells [54,67,75]. Regulatory T cells are cell mediators of peripheral immunological tolerance, and their absence results in excessive multisystem autoimmunity [77,78].

MSCs implantation by different routes has raised very interesting expectations in the treatment of dry eye and regeneration of the ocular surface, thanks to their capacities of immunomodulation and regenerative potential [78,79]. MSC therapy in experimental DES syndrome models improved tear volume and tear film stability, increasing epithelial recovery and the number of goblet cells and decreasing the number of meibomian gland injuries in the conjunctiva [77,80]. Decreasing CD4+ T cells and proinflammatory factors (IL-2, interferon-gamma (IFN-γ), IL-17, and MMP-2) and increasing anti-inflammatory T cell responses augments the regulatory T cells, preventing the progression of the process [80].

Somewhat more than half of human patients with refractory dry eye secondary to chronic graft-versus-host disease (GVHD) treated with MSCs showed reduced symptoms with improved dry eye scores, which suggests that MSCs regulate the balance between Th1 and Th2 [81].

**Figure 1:**
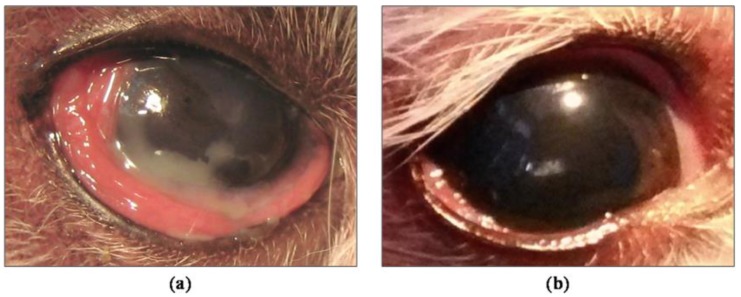
Results of periglandular MSCs (mesenchymal stem cells or multipotent stromal cells) implantation in severe and refractory canine keratoconjunctivitis sicca. (**a**) Before implantation; (**b**) After 9 months of treatment.

Our group was the first to demonstrate the clinical efficacy of periglandular implanted allogeneic MSCs in a canine model. Canines are a superior preclinical animal model, suffering the disease in a natural way similar to humans, avoiding the use of other induced animal models, far from the immune-mediated component of this pathology [82] (Figure 1).

**Figure 2:**
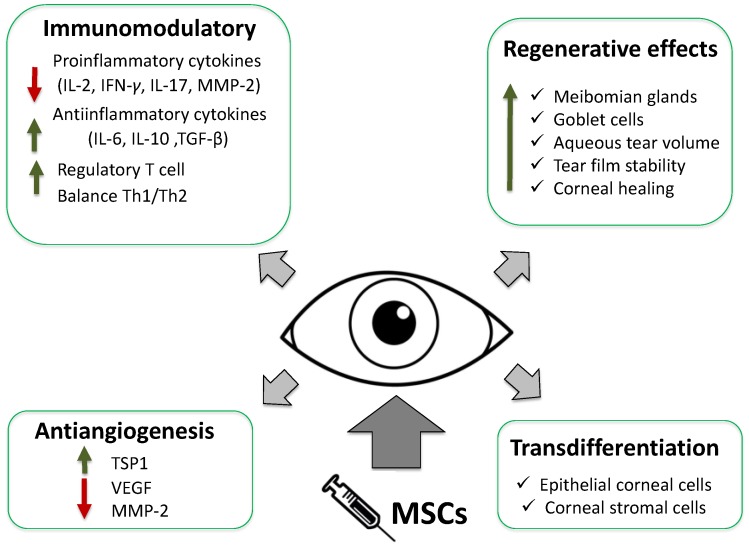
Main mechanisms of action of MSCs implantation in dry eye and ocular surface. IFN-γ: interferon-gamma; IL-2: interleukin 2; MM-2: matrix metalloproteinase-2.

We demonstrated that the MSCs periglandular implantation of the lacrimal glands was an easy and effective system in the functional restoration, with therapeutic response for at least one year, increasing tear secretion and restoring the inherent clinical signs of the disease in dogs refractory to conventional treatment [82]. Similar results have subsequently been reported [83] (Figure 2).

Another interesting aspect was to use allogeneic cells as a therapeutic element. There is increasing evidence that factors such as the age or concomitant diseases affect the efficacy of MSCs [84,85,86]. DED is a pathology that fundamentally affects aged patients and is often accompanied by other immunomediated processes, where the allogeneic therapy presents a great advantage [35,36,37]. This allows the use of perfectly characterized cells in the laboratory from healthy controlled donors and simplifies the logistics for delivery and transplantation [82]. In severe DED, there is damage of the corneal surface [4,9,10]. The damage of the corneal epithelial cell layer and the deeper stromal layer involves a healing process mediated by the activation of progenitor cells that are found in the limbal region of the cornea: the limbal epithelial stem cells (LESCs) [62,78]. Extensive loss of LESCs leads to a persistent corneal epithelial defect and corneal conjunctivalization [79,87].

The importance of the limbal niche in the regeneration of the corneal surface has been perfectly described, where in addition to the existence of a population of limbal epithelial stem/progenitor cells responsible for corneal differentiation, there is a population of MSCs located underneath them with an important role in the regeneration of the corneal surface [62].

Currently LESCs transplantation is the only European Medicines Agency (EMA) authorized cellular therapy for the repair of lesions of the corneal surface. This is possible in unilateral disease, when the contralateral cornea is healthy or less damaged. However, there is a risk of limbal stem cell deficiency development in the donor eye. Allogeneic limbal transplantation has been conducted by a number of ophthalmologists; however, results were not satisfactory, and treatment requires prolonged immunosuppressive therapy [60]. Additionally, the use of MSCs in different models and routes of administration has aroused great expectation in corneal wound healing, decreased conjunctivalization, corneal opacity, inflammation and the area of neovascularization [60,80].

The transplanted MSCs enhanced corneal wound healing by trophic factor production and immune regulatory effect, rather than by direct transdifferentiation into corneal cells [49,62,87]. Corneal scarring is the main complication of corneal wound healing. Corneal fibroblasts (activated stromal keratocytes) migration and their response are modulated by various cytokines and growth factors, which are significantly inhibited by MSCs. The antiscarring capacity of MSCs has been widely reported [49,87].

MSCs have been shown to be good activators of angiogenesis and secrete VEGF [88,89]. However, MSCs seemed to have an opposite effect on corneal angiogenesis, upregulated the expression of thrombospondin-1 (TSP-1, a powerful antiangiogenic factor), and significantly downregulated MMP-2, an inflammation-related proangiogenic factor [90].

In conclusion, based on the multiple effects such as anti-inflammatory, immunomodulatory, antiangiogenic, tear production, and corneal wound healing (reduce neutrophil and macrophage infiltration, help epithelial recovery, decreased number of meibomian gland injuries in the conjunctiva, and increasing number of goblet cells), MSCs are a promising source to treat dry eye syndrome [63,82,90] (Figure 1).

### 3.2. MSCs Secretome

As discussed above, many of the MSCs’ action mechanisms are carried out through the release of a wide variety of bioactive substances encompassed under the concept of the secretome [56,66,70]. The secretome is composed of soluble factors and extracellular vesicles, as microvesicles or exosomes [55,69]. It is thought to be encoded by approximately 10% of the human genome [91,92]. The secretome plays a crucial role as mediators in cell-to-cell interactions and with the surrounding tissues, in functions such as proliferation, differentiation, communication, and migration [56,68].

In recent years, a large number of studies have focused on characterization of the stem cell secretome and have shown great potential in a variety of clinical applications as a cell-free option for regenerative medicine therapies. Ocular surface pathologies are an excellent objective for such a therapy [70,91,92,93,94].

The experimental topical instillation of the secretome in ocular diseases has shown significant improvement in corneal wound healing, attenuating corneal inflammation by inhibition of the proinflammatory cytokines and infiltration of inflammatory cells [94,95]. In allergic conjunctivitis, anti-allergic effects were shown with a reduction of inflammatory cell conjunctival infiltration, inhibition of B cells, mast cells, and histamine functions, through a COX-2-dependent mechanism [96].

The future advantages of the use of the secretome in superficial ocular diseases is to prevent some of the undesirable effects associated with the traditional use of stem cells in regenerative medicine therapy, including reduced concerns for oncogenic potential, lack of immunogenic reaction enabling allogeneic use, and the transmission of infections [68,70]. From the logistic point of view, it could be prepared in advance in large quantities, allowing its long-term storage and immediate delivery for treatment. Stem cells could be considered as tunable pharmacological storehouses useful for combinatorial drug manufacture, delivery, and could be adjusted for different clinical applications [92,96].

## 4. Conclusions

Despite the large number of therapeutic strategies in the management of DED, in recent years new regenerative therapies have consolidated a new perspective in the management of this complex disease, such that it is necessary to have standardization and comparison of their results. This represents an important gateway of hope in the treatment of these pathologies, and an opportunity for further development of pharmacological and cell therapy interventions.

## Figures and Tables

**Table 1:** Comparison of several components between natural tears and different hemoderivatives used in corneal surface.

Component	Tear	AS	PRP	UCS
pH	7.4	7.4	6.61–7.26	7.4
Osmolarity	298–300	296	296	296
Water (%)	98	91	--	--
Albumin (g/dL)	0.39	4–5.3	--	--
Globulins (g/dL)	0.27	2.3	--	--
EGF (ng/mL)	0.2–0.3	0.1–0.2	0.27–4.9	0.5
TGF-β (ng/mL)	2–10	6–33	6.4–67.3	57
NGF (pg/mL)	107.5–468	54–401	37.7	730
IGF-1 (ng/mL)	75.5–157	375	93.5	230
PDGF (ng/mL)	1.33	15–17	13–86	--
VEGF (pg/mL)	--	34.7–160	60–124	--
Vitamin A (ng/mL)	16	372	--	231
Vitamin C (mg/mL)	0.117	0.02	--	--
SP (pg/mL)	69.8–157	71–169	--	245
Lysozyme (mg/mL)	1.4	6	--	6
Surface IgA (µg/mL)	1190	2	--	--
Fibronectin (µg/mL)	21	30–205	28.9–31.1	--
Lactoferrin (ng/mL)	1650	266	--	--
Calcium (mmol/L)	0.3–2	2.5	--	--
Potassium (mmol/L)	26–42	4.5	--	--
Sodium (mmol/L)	120–170	140	--	--

AS: autologous serum; EGF: epidermal growth factor; IGF-1: insulin-like growth factor 1; NGF: nerve growth factor; PDGF: platelet-derived growth factors; PRP: platelet-rich plasma; SP: substance P; TGF-β: transforming growth factor β; UCS: umbilical cord blood serum; VEGF: vascular endothelial growth factor.

## Abbreviations

DED	Dry eye disease
AS	Autologous serum
TGF-β	Transforming growth factor-beta
PDGF	Platelet-derived growth factor
EGF	Epidermal growth factor
NGF	Nerve growth factor
IGF-1	Insulin-like growth factor 1
VEGF	Vascular endothelial growth factor
SP	Substance P
PRP	Platelet-rich plasma
UCS	Umbilical cord blood serum
IL	Interleukin
TNF-α	Tumor necrosis factor-α
CGRP	Calcitonin gene-related peptide
ALS	Allogeneic serum
HGF	Hepatocyte growth factor
PF-4	Platelet factor-4
PRGF	Plasma rich in growth factors
PRPGF	Plasma rich in platelet and growth factors
PC	Platelet concentrate
LR-PRP	Leukocyte-rich platelet-rich plasma
LP-PRP	Leukocyte-poor platelet-rich plasma
MMP-9	Matrix metallopeptidase 9
MSCs	Mesenchymal stem cells or multipotent stromal cells
MHC-II	Major histocompatibility complex II
NK	Natural killer cells
IDO	Indolamine 2,3-dioxygenase
PGE2	prostaglandin E2
NO	Nitric oxide
IFN-γ	Interferon-gamma
LESCs	Limbal epithelial stem cells
TSP-1	Thrombospondin-1
